# Volatile Characterization of Lychee Plant Tissues (*Litchi chinensis*) and the Effect of Key Compounds on the Behavior of the Lychee Erinose Mite (*Aceria litchii*)

**DOI:** 10.3390/biom13060933

**Published:** 2023-06-02

**Authors:** Livia M. S. Ataide, Nurhayat Tabanca, Maria A. Canon, Elena Q. Schnell, Teresa I. Narvaez, Kevin R. Cloonan, Paul E. Kendra, Daniel Carrillo, Alexandra M. Revynthi

**Affiliations:** 1 Tropical Research and Education Center, University of Florida, 18905 SW 280 ST, Homestead, FL 33031, USA; malejandra1@ufl.edu (M.A.C.); dancar@ufl.edu (D.C.); 2 Subtropical Horticulture Research Station, U.S. Department of Agriculture, Agricultural Research Service (USDA-ARS), 13601 Old Cutler Road, Miami, FL 33158, USA; nurhayat.tabanca@usda.gov (N.T.); elena.schnell@usda.gov (E.Q.S.); isabelt87@hotmail.com (T.I.N.); kevin.cloonan@usda.gov (K.R.C.); paul.kendra@usda.gov (P.E.K.)

**Keywords:** herbivore-induced plant volatiles (HIPVs), behavioral bioassays, eriophyoid mites, gas chromatography-mass spectrometry (GC-MS), head-space-solid phase microextraction (HS-SPME)

## Abstract

Herbivore-Induced Plant Volatiles (HIPVs) are volatile signals emitted by plants to deter herbivores and attract their natural enemies. To date, it is unknown how lychee plants, *Litchi chinensis*, respond to the induction of leaf galls (erinea) caused by the lychee erinose mite (LEM), *Aceria litchii*. Aiming to reveal the role of HIPVs in this plant-mite interaction, we investigated changes in the volatile profile of lychee plants infested by LEM and their role on LEM preferences. The volatile profile of uninfested (flower buds, fruit, leaves and new leaf shoots) and infested plant tissue were characterized under different levels of LEM infestation. Volatiles were collected using head-space-solid phase microextraction (HS-SPME) followed by gas chromatography-mass spectrometry (GC-MS) analyses. Fifty-eight volatiles, including terpenoids, alcohols, aldehydes, alkanes, esters, and ketones classes were identified. Using dual-choice bioassays, we investigated the preference of LEM to uninfested plant tissues and to the six most abundant plant volatiles identified. Uninfested new leaf shoots were the most attractive plant tissues to LEM and LEM attraction or repellence to volatiles were mostly influenced by compound concentration. We discuss possible applications of our findings in agricultural settings.

## 1. Introduction

Plants have sophisticated defense systems to recognize and counterattack herbivorous mites and insects. Such defensive responses involve changes in plant traits that interfere with herbivores directly or indirectly. Direct defenses include the production of toxicants and volatile organic compounds (VOCs) that repel or deter herbivores, while indirect defenses include the production of VOCs that attract natural enemies of the herbivore (for reviews of VOCs as direct and indirect defenses see Howe et al. [1] and Dudareva et al. [2] and the references therein). VOCs with toxic, repellent, or attractive properties include various groups of terpenoids (monoterpenes, sesquiterpenes and homoterpenes), phenylpropanoids and benzenoids, fatty acid derivatives, and amino acid derivatives [3,4,5]. Upon mechanical damage, mite, or insect attack, the plants’ immune responses trigger the production of novel VOCs, known as Herbivore-Induced Plant Volatiles (HIPVs), locally and systemically within the plant [4]. 

The most well-documented function of HIPVs is the attraction of natural enemies [4,5,6], which indirectly promotes plant fitness [7]. Several studies have shown that terpenoids and other VOCs, emitted in response to herbivory, allow parasitoids and predators to distinguish infested from non-infested plants, helping them locate their prey. For instance, Lima bean [8], apple [9], and cucumber [10] plants infested with the two-spotted spider mite (*Tetranychus urticae*, Acari: Tetranychidae) attracted significantly more natural enemies than non-infested plants. The composition of HIPVs can vary significantly within plant species when attacked by the same herbivore. For example, feeding by *T. urticae* induces different HIPV blends from a range of host plants [11], and the predatory mite *Phytoseiulus persimilis* (Acari: Phytoseiidae) is attracted to different HIPV blends from different *T. urticae* -damaged plant species [12]. 

In addition to directly repelling herbivores [13] and indirectly attracting natural enemies, HIPV’s are also involved in within-plant communication [14,15] and plant-plant communication in response to herbivore attack [16,17,18,19]. Herbivory releases distinct volatile blends in damaged plants that are potent elicitors of defenses in systemic uninduced leaves of the same plant or nearby plants [20,21,22]. Since HIPVs can be used to recruit natural enemies and repel herbivores, they can be implemented as a sustainable Integrated Pest Management (IPM) strategy for controlling plant pests. Furthermore, transgenic plants can be engineered to enhance emission of VOCs to increase attraction of natural enemies [23,24,25,26] and repel important pests [27,28]. 

Lychee (*Litchi chinensis* Sonn., Sapindaceae) is a commercially important fruit native to Asia that has been cultivated by several countries in the tropical and subtropical regions of the world [29]. A tiny eriophyoid mite called the lychee erinose mite (LEM), *Aceria litchii* (Keifer) (Acari: Eriophyidae), threatens lychee production worldwide as it infests every plant tissue. LEM is highly host-specific and is only known to attack lychee [30,31]. Upon feeding, LEM induces the formation of a gall called “erineum” which consists of proliferations of hypertrophic trichomes [32] that undergo several stages of development. In early erineum stages, when mite infestations are low, the affected tissue becomes distorted, and white hairs develop as feltlike masses. As mite populations grow, a denser white erineum becomes apparent, and when populations peak, the erineum turns into an amber color. At its final stage, the erineum turns dark in color followed by tissue necrosis [30]. Erinea can be induced in several plant tissues that have not hardened yet, such as new leaf shoots, petioles, flower buds, and fruitlets [32]. LEM management has focused on removing LEM infested plant parts by pruning [33,34] followed by repeated pesticide (sulfur) applications to protect the new shoots [32,35]. However, these strategies are insufficient to mitigate LEM damage, which can reduce yields by up to 80% [36,37].

To date, it is unknown how and why LEM induces the formation of erinea. Recently, a growing body of literature shows that the erinea are a perfect environment for LEM to hide and acquire protection from biotic and abiotic stressors. For instance, pesticides eliminated nearly all LEM when applied directly [38], but not when mites were sheltered inside the erinea [39]. Moreover, although several predatory mites have been found associated with LEM erinea [40,41,42,43] evidence is lacking on their ability to prey on mites inside the erinea. Hence, control methods focused on forcing the mites to exit the erinea to target them with pesticides or natural enemies can contribute to managing this mite pest. In addition, it is unknown what drives the mites to disperse to new young tissues within and among plants. One possibility is that volatile compounds emitted by immature lychee tissues may trigger their initial dispersal. 

Previous studies have explored the odor and flavor of lychee fruits, and described them as honey, rose-floral and citrus-fruity [44,45]. Volatiles emitted by fresh fruit [44,46,47], leaves [48], juices [44,49], wines [50] and canned fruit [51] of several lychee cultivars have been characterized. Although the volatile extraction methods varied among different studies, all reported terpenoids and alcohols as major volatile components of lychee fruit [44,45,47,52,53] and young and old lychee leaves [48]. Recently, Gunpal and Patni [54] compared methanolic extracts from non-infested and LEM-infested lychee leaflets to determine which metabolic processes of the leaf were altered by LEM. Leaf extracts were rich in sugars, terpenoids, linoleic acid esters, vitamins, antioxidants, glycerol, and steroids. However, it remains to be investigated which VOCs are emitted by lychee tissues distinctly targeted by LEM, i.e., new leaf shoots, leaves, flower buds, and fruitlets, and which HIPVs are synthesized by the plant after LEM infestation. In addition, LEM preferences to lychee VOCs or HIPVs are unknown and their preferences for specific plant tissues have not been studied.

In line with this goal, laboratory bioassays were performed to assess the importance of volatile compounds in the LEM-lychee interaction. First, the role of VOCs was investigated by characterizing volatile profiles of uninfested lychee plant tissues (floral buds, open flowers, leaves, and new leaf shoots) and LEM’s preference for them. Second, potential HIPVs were assessed by identifying volatiles of plants with different levels of LEM infestation (initial, intermediate, heavy, and overexploited). Third, dual-choice bioassays were carried out to investigate LEM’s attraction or repellence to the most abundant lychee volatiles. The end goal of this study was to address the role of the key VOCs and HIPVs in LEM behavior. This information may help to develop management strategies that force the mites to leave the erinea and/or lure natural enemies to LEM infested plants. 

## 2. Materials and Methods

### 2.1. Plant Material and LEM Infestation

Lychee plants (var. ‘Mauritius’) were grown from seed in pots 3.7 L filled with ProMix (ProMix BX Mycorhizae, Denver, CO, USA) and kept in a climate room (25 ± 2 °C, 50% RH, and a 12:12 h (L:D) photoperiod) in the biocontainment facility of the Tropical Research and Education Center (TREC), University of Florida, Homestead, FL (25.50° N, 80.49° W) under the Florida Department of Agriculture and Consumer Services, Division of Plant Industry permit 2018-029. The plants were watered twice per week and fertilized every 15 d with 24-8-16 (N-P-K) (Miracle-Gro^®^, The Scotts Company, Marysville, OH, USA) and 138 chelated EDDHA iron (Sequestrene^®^, Syngenta, Wilmington, DE, USA). LEM-infested leaflets were detached from the infested lychee plants collected from the FruitScapes Nursery (Bokeelia, FL, USA; 26.38° N 82.07° W) and placed on 4-month-old pest-free lychee seedlings showing new leaf shoots. Mite establishment was confirmed by appearance of erineum which varied per plant from 6 to 30 days. Erineum development was assessed by the change of color and trichome density. The density of the erineum and its color seem to be two important factors shaping mite population sizes among stages, varying from zero, in the initial infestation, to thousands, in the heavy infestation stage (Ataide et al. in preparation). To obtain erinea of different colors available for the experiments, the lychee plant infestation was repeated four times using plants of the same age. Infested plants were separated into four groups, covering four erinea stages, hereafter called initial infestation, intermediate infestation, heavy infestation, and overexploitation. Thus, five treatments were conducted for potted (infested) lychee plants (A): initial infestation (A1), intermediate infestation (A2), heavy infestation (A3), plant overexploitation (A4), and healthy, uninfested plant (A5) (Figure S1). In addition, uninfested lychee branches were cut from fully grown lychee trees ‘Mauritius’ at TREC and USDA-ARS Subtropical Horticulture Research Station, Miami, FL (25.64° N, 80.29° W). From these floral buds, open flowers, leaf buds and new leaf shoots were obtained. Thus, four treatments were conducted for field-collected (non-infested) lychee tissues (B): floral buds (B1), open flowers (B2), leaf buds (B3), and new leaf shoots (B4). LEM dual-choice bioassays and volatile collections were carried out at TREC’s biocontainment facility using potted plants and field-collected tissues, separately (see below).

### 2.2. Chemical Standards

Chemical standards were purchased from the following sources: (Z)-3-hexen-1-ol (Cas 928-96-1), α-pinene (Cas 80-56-8), sabinene (Cas 3387-41-5), β-pinene (Cas 127-91-3), 6-methyl-5-hepten-2-one (Cas 110-93-0), (+)-limonene (Cas 5989-27-5), mixture of ocimene isomers (Cas 13877-91-3), γ-terpinene (Cas 99-85-4), linalool oxide (Cas 60047-17-8), terpinolene (Cas 586-62-9), linalool (Cas 78-70-6), nonanal (Cas 124-19-6), estragole (Cas 140-67-0), decanal (Cas 112-31-2), (+)-cyclosativene (Cas 22469-52-9), tetradecane (Cas 629-59-4), methyl eugenol (Cas 93-15-2), β-caryophyllene (Cas 87-44-5), aromadendrene (Cas 489-39-4), α-humulene (Cas 6753-98-6), a mixture of farnesene isomers (Cas 502-61-4), pentadecane (Cas 629-62-9), hexadecane (Cas 544-76-3), heptadecane (Cas 629-78-7) from Sigma-Aldrich, St. Louis, MO, USA; α-copaene (Cas 3856-25-5) from Fluka Chemical Co., Buchs, SG, Switzerland), and (+)-*ar*-curcumene (Cas 4176-06-1), from BOC Sciences Shirley, NY, USA.

### 2.3. Headspace Volatile Collections

Volatiles were collected from potted plants (A) and field-collected tissues (B) from fully-grown trees of the same (‘Mauritius’) cultivar. Each potted plant (A1 to A5) was enclosed in an oven bag (406 mm × 444 mm, Reynolds, Richmond, VA, USA) and sealed with a cotton strip on the upper 5 cm above soil level to avoid soil emissions. Prior to use, oven bags were baked in an oven at 120 °C for 2 h, and inflated with clean air and deflated three times to remove any residual contamination [55]. Bags were then stored in the laboratory until use. VOCs from the lychee plants were collected by head-space-solid phase microextraction (HS-SPME). The medium polarity SPME fiber, polydimethylsiloxane/divinylbenzene (PDMS/DVB, 65 µm) (Supelco, Bellefonte, PA, USA) was used to extract volatile emissions from the samples. Before use, the SPME fiber was conditioned for 30 min at 225 °C in the gas-chromatograph injection port under the carrier gas, helium. 

Volatiles from the potted plants were allowed to accumulate within the airspace of the bag for 10 min to equilibrate. The SPME device was affixed to a stand with a clamp. The fiber was introduced through the bag and exposed for 24 h at room temperature. After extraction, the fiber was removed from the sample; subsequently, the fiber was inserted into the injector port for gas chromatography-mass spectrometry (GC-MS) analysis. The desorption of the volatile compounds was performed at 220 °C for 2 min. Nine replicates were performed on different days. Blank measurements with empty oven bags and fibers were performed before each analysis to prevent the release of undesirable compounds. For field-collected samples (B1 to B4), a 30 mL beaker containing 1.003 g (±0.02 g) of each plant part was covered with aluminum foil and then subjected to saturate the headspace with the volatiles for 15 min at room temperature. Subsequently, a preconditioned fiber was pushed through the film and exposed to the headspace of each plant tissue for 30 min. Thereafter, volatile compounds were desorbed in the GC-MS injector at 220 °C for 2 min. Each plant sample was extracted by HS-SPME and GC-MS in three replicates. The blank SPME fiber was run before sampling to ensure that it was free of contaminants. 

### 2.4. Gas Chromatography-Mass Spectrometry Analysis (GC-MS)

Volatile emissions were identified by gas chromatography/mass spectrometry (GC/MS) coupled to a 5975B quadrupole mass spectrometer (Agilent Technologies, Santa Clara, CA, USA) equipped with a DB-5 column (30 m × 0.25 mm × 0.25 µm). After sampling, the SPME fiber was introduced onto the GC injector in the splitless mode under the following conditions. The oven temperature program was set at 60 °C for 1.3 min and increased to 246 °C at 3 °C/min; the total time was 63.3 min. The carrier gas helium was set at 1.3 mL/min. The injector temperature was set at 220 °C and the detector temperature was 230 °C. Electron ionization mode (EI-MS; 70 eV) was used to acquire mass spectra in the 35-550 *m*/*z* at 2.8 scans/s. MassHunter software (B.07.06, Agilent Technologies, Santa Clara, CA, USA) was utilized to obtain the mass spectra and ion chromatogram. Volatile emissions of lychee plants were identified by a combination of 3 methods: (1) The individual peaks were identified by comparing their mass spectra with Wiley 12/National Institute of Standards and Technology (NIST) [56], MassFinder [57], Adams Library [58], Flavors and Fragrances of Natural and Synthetic Compounds 3 [59]; (2) in-house library “SHRS Essential Oil Constituents-DB-5” which was built up from authentic standards purchased from different sources, and components of known essential oils analyzed under the same conditions [60]; (3) to confirm the identification, retention index (RI) values calculated according to the RI van Den Dool and Kratz equation RI_x_ = 100_n_ + 100 (t_x_ − t_n_)/(t_n+1_ − t_n_); t_n+1_ and t_n_ retention times of the reference *n*-alkane hydrocarbons eluting immediately before and after compound “X”; t_x_ retention time of compound “X” [61] to a series of *n*-alkanes (C_9_–C_21_, Sigma-Aldrich, St. Louis, MO, USA), and compared with their RIs available in the literature [58,62,63,64]. The relative percentages were obtained by total ion current (TIC) peak areas.

Principal Component Analysis (PCA) and Agglomerative Hierarchical Clustering (ACH) for GC-MS data were performed using XLSTAT 2021 (Addinsoft, New York, NY, USA). Only constituents in a concentration higher than 0.5% were used as variables for the PCA and ACH analysis. Data used for the PCA and ACH analyses were a 21 × 5 matrix (105 data) for samples A1 to A5, and 81 × 4 matrix (112 data) for samples B1 to B4. Pearson’s correlation model was used for PCA. Euclidean distance for measure and Ward’s method were used for ACH analysis.

### 2.5. Dual-Choice Test between Field-Collected (Non-Infested) Lychee Tissues

**Figure 1:**
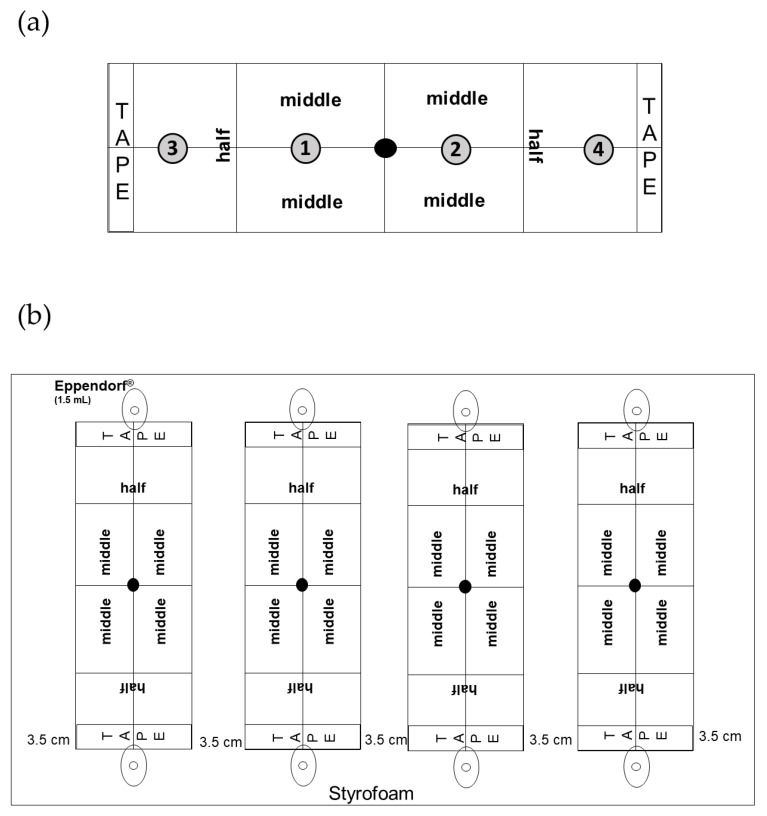
Schematic drawing of the bioassay used for assessing LEM preferences. Panel (**a**) shows the experimental arena used in the dual-choice bioassay. The drawing represents a microscope slide (75 mm × 25 mm) divided into Sections 1–4 and TAPE. Panel (**b**) shows the white Styrofoam board (20 cm × 10 cm) used to hold four slides and two Eppendorf^®^ tubes (1.5 mL) per slide. Only LEM crossing half of the slide (Sections 3 and 4 and TAPE) were considered to make a choice between the two sources offered. The number of mites in each section was assessed after 24 h.

Considering the minute size of LEM (~200 nm), it was not possible to carry out conventional dual-choice bioassays using Y-tube olfactometers [65], petri-dish arenas [66], cages or greenhouses [67], as used for other mite species. All known experimental set-up were tested, but without yielding any mite response. Therefore, a new method to conduct dual-choice bioassays was developed. The experimental arena consisted of a microscope glass slide (75mm × 25mm) with markings dividing it into four equal sections (Figure 1a). Double-sided tape (1.25 cm wide) (Scotch^®^, 3M, Miami, FL, USA) was placed alongside the edges of the slide to keep the mites in the arena (Figure 1a). Then, an Eppendorf^®^ tube filled with nutrient solution (10N-5P-14K) (MaxiGro^®^, General Hydroponics Inc., Sebastopol, CA, USA) and holding one plant tissue (flower buds, fruits, leaves, or new leaf shoots), was placed on two opposite sides of the arena. Four arenas were placed on a white Styrofoam board (20 × 10 cm) (Figure 1b) and each board was placed inside a custom-made acrylic cage (30 × 30 × 30 cm) with openings on two sides to allow airflow. The experimental units were kept at 25 ± 2 °C, 50% ± 10% RH, and a 12:12 h (L:D) photoperiod. Fluorescent lamps were placed above each cage to provide uniform light over the experimental arenas. A leafdisc (0.5 cm diam.), cut from an amber-colored erinea (i.e., fully infested with LEM) was placed in the middle of each arena (on the black dot Figure 1a), and after 24 h, the mites that moved towards one side and crossed the midline marking were considered attracted to the plant part offered on the same side. To test whether LEM was able to make reliable choices we offered the same plant tissue on both edges, as controls. The plant tissues tested were leaf, new leaf shoot, fruit, and quiescent flower bud. All possible combinations of plant tissues were offered to LEM in a factorial design (*N* = 8). Only LEM found in Sections 3 and 4 and TAPE (Figure 1a) were considered in the analyses. Differences in the proportion of mites between treatments were tested with generalized linear mixed-effects models (GLMM) using the *lme4* package [68]. Binomial error distribution and including the position of the slide (left or right) and Styrofoam board (replicate) as a random factor [69]. Residual plots of data revealed no major deviation from the normality and variance homogeneity assumptions. Analyses were performed separately for each plant tissue/combination and using the R program version 4.2.1 [70].

### 2.6. Dual-Choice Test on Single Volatile Compounds

Using the same experimental unit as above, dual-choice bioassays were used to evaluate LEM attraction to the six major volatile compounds identified in potted plants and field-collected tissues (nonanal, decanal, sabinene, limonene, β-caryophyllene and *ar*-curcumene; see Result Section 3.1). Eight concentrations were tested for each compound: 3%, 5%, 7%, 10%, 25%, 50%, 75% and 100%. Compounds were diluted in absolute EtOH, which was also used as a control. All dual-choice tests were made by offering a single compound concentration against the control (EtOH). To test whether LEM was able to make reliable choices we offered the clean filter paper or ethanol on both sides, as controls. Forty-five minutes prior to testing, 5 μL of each compound was applied to a circular filter paper (d = 1 cm), allowing enough time for the EtOH to evaporate. Then, the filter paper containing the test compound was placed on one end of the slide, and the filter paper containing EtOH (control) was placed on the other end (Figure 1a). The assessment of LEM choice between the two odor sources was performed as above. Replicates in which LEM made no choice (remained in the middle of the arena, Sections 1 and 2) were discarded. To correct for the potential positional bias, the treatment and the control sources were switched from left to right in all choice experiments. The number of replicates per compound concentration are shown in their respective figures (see Section 3). The same analysis described above was performed separately for each compound, as well as each concentration. 

## 3. Results

### 3.1. Volatile Composition of Potted (Infested) Lychee Plants and Field-Collected (Non-Infested) Lychee Tissues

**Figure 2:**
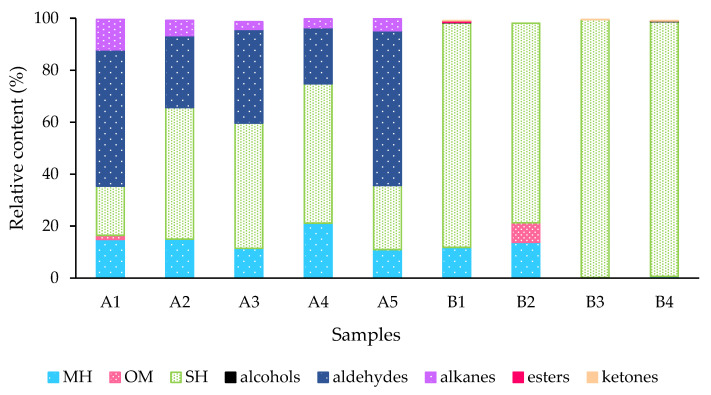
Abundance (%) of the chemical classes in volatiles from potted (infested) lychee plants and from field-collected (non-infested) lychee tissues. MH- monoterpene hydrocarbons, OM- oxygenated monoterpenes, SH- sesquiterpene hydrocarbons, alcohols, aldehydes, alkanes, esters, and ketones. Initial infestation (A1), intermediate infestation (A2), heavy infestation (A3), overexploitation (A4), and healthy, uninfested plant (A5). Floral buds (B1), open flowers (B2), leaf buds (B3), and new leaf shoots (B4).

A total of 58 compounds were identified from potted lychee plants, accounting for 98.05% to 99.73% of total volatiles: 44 terpenoids, 2 esters, 1 alcohol, 1 ketone, 4 alkanes, and 6 aldehydes (Table 1). In lychee leaves with different LEM infestation levels (A1 to A5), monoterpene hydrocarbons (MHs), sesquiterpene hydrocarbons (SHs), aldehydes and alkanes were the major group of volatile components (Figure 2). Among the volatile terpenoids in samples analyzed, sabinene (1.92% to 11.67%), limonene (3.81% to 9.94%), β-caryophyllene (5.21% to 10.34%), *ar*-curcumene (10.25% to 38.54%) were the most abundant compounds, followed by α-copaene (1.08% to 2.82%) and α-zingiberene (0.24% to 2.03%). The aliphatic aldehydes were most characterized by higher relative amounts of nonanal (18.65% to 51.52%) and decanal (2.28% to 7.76%). Nonanal was more abundant in stage A1 (43.52%), followed by 24.06% in stage A2, 29.31% in stage A3, and 18.65% in stage A4. Nonanal concentration was higher in control plants (A5, 51.52%), and A1 stage plants, than in plants under higher levels of infestation (A2, A3, A4). A similar pattern was observed for decanal, 7.76% in control plants and 6.74% in stage A1. (*E*)-2-decanal (1.58%), undecanal (0.30%), and (*E*)-2-undecanal (0.12%) were higher in stage A1 than in other stages. Similar patterns were also observed with alkanes. Hexadecane (2.21%) and heptadecane (5.38%) were found in higher concentrations in the initial infestation stage than in the later infestation stages. The concentration of pentadecane was higher in early infestation stages (0.82% to 1.03%) than in progressed (0.21%) and over-exploitation stages (0.37%). 

Regarding the volatiles from field-collected tissues (B1 to B4), sesquiterpene hydrocarbons were the highest among all categories and represented approximately 77% to 99% of the total volatile profile in flower buds (86.44%), open flowers (76.81%), leaf buds (99.25%), and new leaf shoot (98.02%, Figure 2). Major SHs identified were zingiberene (16.50% to 39.62%), *ar*-curcumene (11.64% to 26.50%), β-caryophyllene (10.57% to 13.98%), γ-muurolene (4.26% to 7.70%), β-sesquiphellandrene 2.02% to 5.94%), α-copaene (2.04% to 2.86%), α-humulene (0.64% to 2.19%) and *trans*-α-bergamotene (1.15% to 2.15%). Monoterpene hydrocarbons (MHs), α-thujene (1.71% and 1.2%), α-pinene (1.99% and 1.45%), sabinene (6.49% and 4.23%), and (*E*)-β-ocimene (0.59% and 5.74%) were present mostly in flower buds and open flowers, respectively, while β-pinene (0.43%) was only found in open flowers. Limonene was about 5.5 times more abundant in open flowers than flower buds and new leaf shoots. From the group of oxygenated monoterpenes (OMs), OM linalool and its *cis*- and *trans*-linalool oxides (furanoid) were only detected in open flowers (Table 1). 

**Figure 3:**
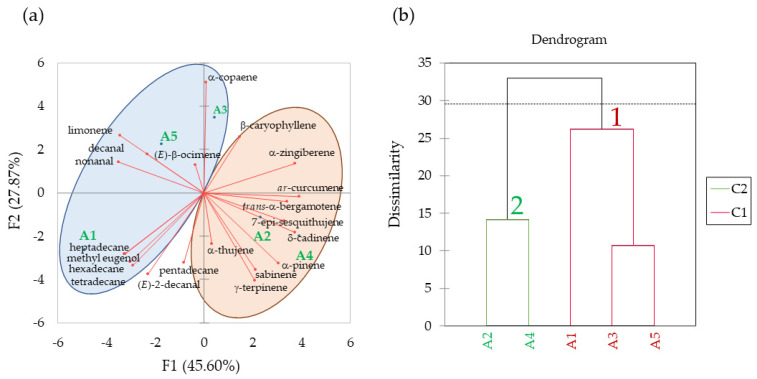
Panel (**a**) shows the Biplot Analysis for Principal Component Analysis (PCA) of volatile compounds emitted from potted lychee plants under different levels of LEM infestation (A1 to A5). Panel (**b**) shows the Agglomerative Hierarchical Cluster (AHC) analysis for LEM infestation samples A1 to A5 based on selected volatile active compounds. Initial infestation (A1), intermediate infestation (A2), heavy infestation (A3), overexploitation (A4), and healthy, uninfested plant (A5).

**Figure 4:**
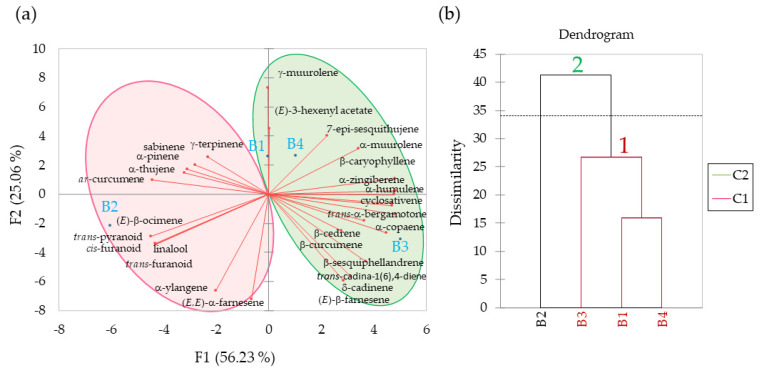
Panel (**a**) shows the Biplot Analysis for Principal Component Analysis (PCA) of volatile compounds emitted from field-collected (non-infested) lychee tissues (B1 to B4). Panel (**b**) shows the Agglomerative Hierarchical Cluster (AHC) analysis for samples B1 to B4 based on selected volatile active compounds. Floral buds (B1), open flowers (B2), leaf buds (B3), and new leaf shoots (B4).

Principal component analysis (PCA) was carried out to identify the grouping pattern among potted (infested) lychee plants (Figure 3) and field-collected (non-infested) samples (Figure 4) based on volatile compounds obtained from GC-MS data (Table 1). For better distinction between the groups, we focused on the proportions of the volatile compounds. Based on the Eigenvectors criterion, principal components with an eigenvalue greater than one are considered important. First, PCA analysis was applied based on the GC-MS data from samples A1 to A5. Principal components F1 and F2 had higher Eigenvectors of 9.58 and 5.85, respectively (Table 2). Two macro principal components accounted for 45.60% (F1) and 27.87% (F2) representing 73.47% of the total variance, indicating that the F1 and F2 had good support for the difference in the plant tissues (Figure 3a). 

Table 3 shows the percent contribution and squared cosine values (cos^2^) of each principal component. The percent contribution of each variable shows the most important variables. Also, the cos^2^ values indicate the potential importance of components for a given observation. Taking the values of cos^2^ and the percent contribution, nonanal (7.63%; 0.73), decanal (7.44%; 0.71), *ar*-curcumene (9.57%; 0.92), α-zingiberene (8.59%; 0.82) and δ-cadinene (8.60%; 0.82) showed a strong impact on the F1, while α-copaene (16.13%; 0.94) showed the strongest impact on the F2. Cos^2^ for F1 was the highest impact on the observation of initial infestation A1 (0.758) and overexploitation A4 (0.614); heavy infestation A3 (0.724) was the highest impact on F2. 

Agglomerative Hierarchical Clustering (AHC) shows the similarity between existing and merged clusters. Different LEM infestation levels were classified into 2 clusters (Figure 3b). Cluster 1 includes initial infestation (A1), heavy infestation (A3), and uninfested plant (A5), and the second cluster included intermediate infestation (A2) and overexploitation (A4). Compounds limonene, nonanal, decanal, β-caryophyllene, *ar*-curcumene showed a strong impact on cluster 1, while compounds α-pinene, sabinene, limonene, β-caryophyllene, *ar*-curcumene showed a strong impact on the cluster 2 (Table 4).

PCA analysis was applied based on the GC-MS data from samples B1 to B4. The results showed three best eigenvectors of 15.74 (F1), 7.02 (F2), and 5.24 (F3), describing 56.23%, 25.06%, and 18.71% of the total variance, respectively (Table 5). Most variables were accumulated at 81.29% in the principal components F1 and F2 (Figure 4a).

Cos^2^ values from field-collected (non-infested) lychee tissues (B1 to B4) on the F1 axis were strongly correlated with (*E*)-β-ocimene (0.84), cyclosativene (0.92), α-copaene (0.96), β-caryophyllene (0.97), *trans*-α-bergamotene (0.92), α-humulene (0.95), *ar*-curcumene (0.82), α-zingiberene (0.97) and β-sesquiphellandrene (0.84), which contributed 5.34%, 5.85%, 6.12%, 6.15%, 5.85%, 6.03%, 5.23%, 6.19%, and 5.34%, respectively, while cos^2^ values on the F2 axis strongly correlated with α-ylangene (0.98), γ-muurolene (0.99), (*E*,*E*)-α-farnesene (0.82), which contributed 13.90%, 14.16%, and 11.64%, respectively (Table 6).

AHC was performed on the volatile composition obtained from field-collected samples B1 to B4. Samples of floral buds (B1), leaf buds (B3), and new leaf shoots (B4) were grouped into the same cluster, in agreement with the PCA results, while the open flower sample (B2) was clustered alone (Figure 4b). Maximum distance to centroid was found for cluster 1 of 8.73 between two clusters, indicating the similarity of chemical profiles among the B1, B3 and B4 samples (Table 7). Cluster 2 showed maximum distances to centroid was zero, indicating it was closed to cluster 1. Compounds α-zingiberene, *ar*-curcumene and β-caryophyllene showed a strong impact on cluster 1 and 2. This finding was also in accordance with the experimental data detected by GC-MS (Table 1). 

### 3.2. LEM Behavioral Responses to Field-Collected (Non-Infested) Lychee Tissues and Single Volatile Compounds

**Figure 5:**
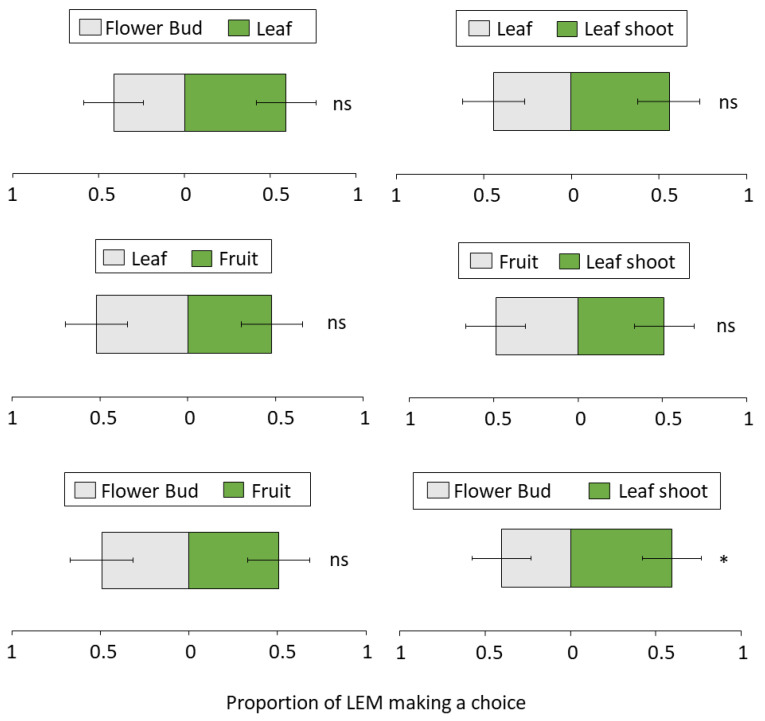
Preference of LEM when offered different field-collected (non-infested) lychee tissues. Panel shows LEM choice when offered different combinations of plant tissues (leaf vs. fruit, leaf vs. bud, leaf vs. leaf shoot, fruit vs. bud, fruit vs. leaf shoot, leaf shoot vs. leaf shoot, *N* = 8). * GLMM: *p* < 0.05; ns, not significant. Bars indicate proportion (±SE) of mites reaching the end section of the slide (Sections 3 and 4 and TAPE) towards each plant tissue in 24 h.

In almost all cases, when given a choice between two field-collected (non-infested) lychee tissues (control groups), there was no significant difference in the number of LEM that reached both sources. Except when presented with a choice between leaf shoot vs. leaf shoot there was a slightly significant difference (*p* = 0.04) towards one of the sides (Figure S2). For the other groups tested, fruit vs. fruit (*p* = 0.1), leaf vs. leaf (*p* = 0.1) and bud vs. bud (*p* = 0.31) there was no difference between choices made by LEM. When different plant tissues were offered, LEM showed a preference for leaf shoot over buds (*p* = 0.04, Figure 5). No clear choice was observed for the other treatments, leaf vs. fruit (*p* = 0.61), leaf vs. bud (*p* = 0.12), leaf vs. leaf shoot (*p* = 0.31), fruit vs. bud (*p* = 0.31) and fruit vs. leaf shoot (*p* = 0.1).

**Figure 6:**
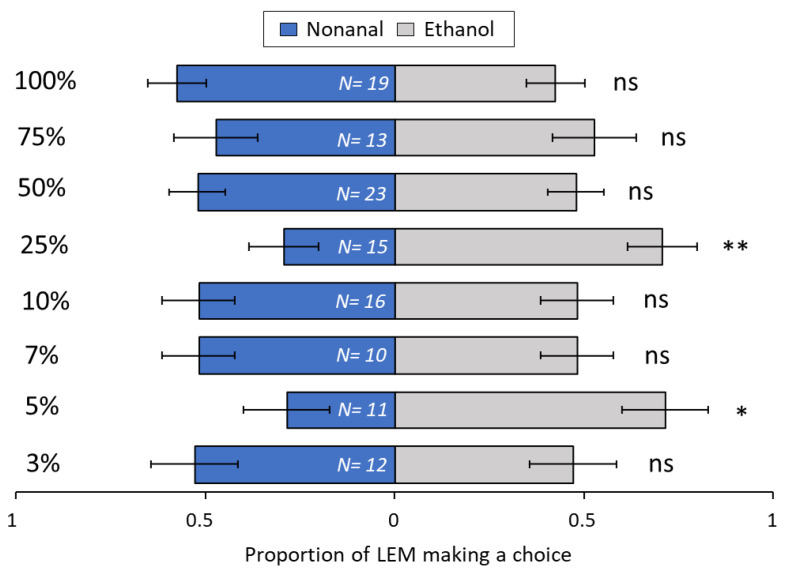
Preference of LEM to the volatile compound nonanal and its solvent ethanol (control), in a dual-choice experimental unit. Panel shows eight Nonanal concentrations tested: 3%, 5%, 7%, 10%, 25%, 50%, 75% and 100%. Numbers inside bars represent number of replicates; GLMM: * *p* < 0.05; ** *p* < 0.01; ns, not significant. Bars indicate the proportion (±SE) of mites reaching the end section of the slide towards nonanal and ethanol in 24 h.

**Figure 7:**
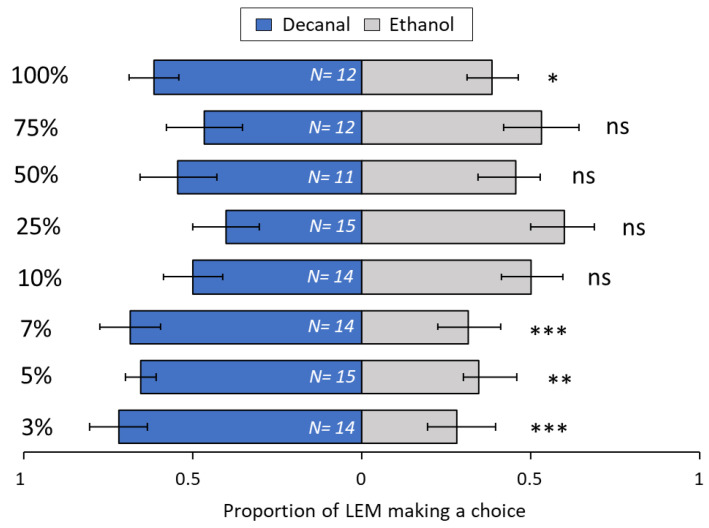
Preference of LEM to the volatile compound decanal and its solvent ethanol (control), in a dual-choice experimental unit. Panel shows all eight Decanal concentrations tested: 3%, 5%, 7%, 10%, 25%, 50%, 75% and 100%. Numbers inside bars represent number of replicates; GLMM: * *p* < 0.05; ** *p* < 0.01, *** *p* < 0.001; ns, not significant. Bars indicate proportion (±SE) of mites reaching the end section of the slide towards decanal and ethanol in 24 h.

In dual-choice assays between single volatile compounds, the concentration was the most important factor determining LEM attraction or repellence. The most repellent compound, aliphatic aldehyde nonanal (Figure 6), repelled over 70% of LEM at 5% (*p* = 0.02) and 25% nonanal (*p* = 0.003) concentrations. For the other concentrations of nonanal, 3% (*p* = 0.10), 7% (*p* = 0.65), 10% (*p* = 0.28), 50% (*p* = 0.13), 75% (*p* = 0.10) and 100% (*p* = 0.17), LEM choice was not significantly different between nonanal and the solvent control (Ethanol). Several concentrations of aliphatic aldehyde decanal (Figure 7), were attractive to LEM: 3% (*p* ≤ 0.001), 5% (*p* = 0.009), 7% (*p* ≤ 0.001) and 100% (*p* ≤ 0.04). However, no choice was observed between the control and decanal at 10% (*p* = 0.69), 25% (*p* = 0.14), 50% (*p* = 0.1) and 75% (*p* = 0.68).

**Figure 8:**
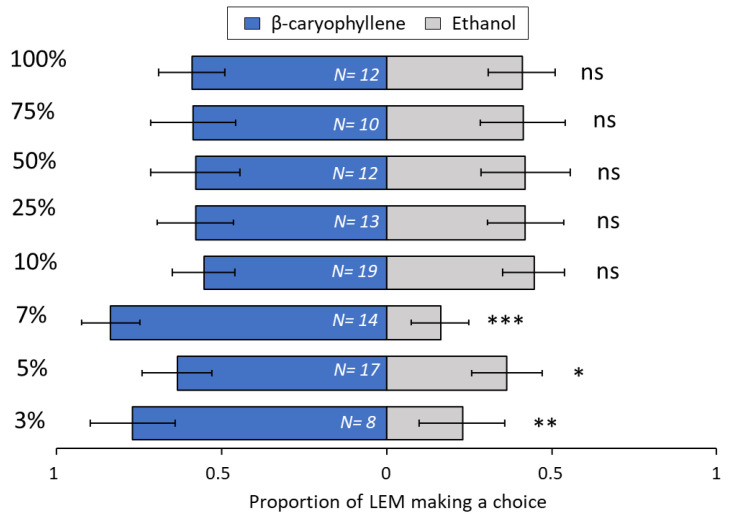
Preference of LEM to the volatile compound β-caryophyllene and its solvent ethanol (control), in a dual-choice experimental unit. Panel shows all eight β-caryophyllene concentrations tested: 3%, 5%, 7%, 10%, 25%, 50%, 75% and 100%. Numbers inside bars represent number of replicates; GLMM: * *p* < 0.05; ** *p* < 0.01, *** *p* < 0.001; ns, not significant. Bars indicate proportion (±SE) of mites reaching the end section of the slide towards β-caryophyllene and ethanol in 24 h.

**Figure 9:**
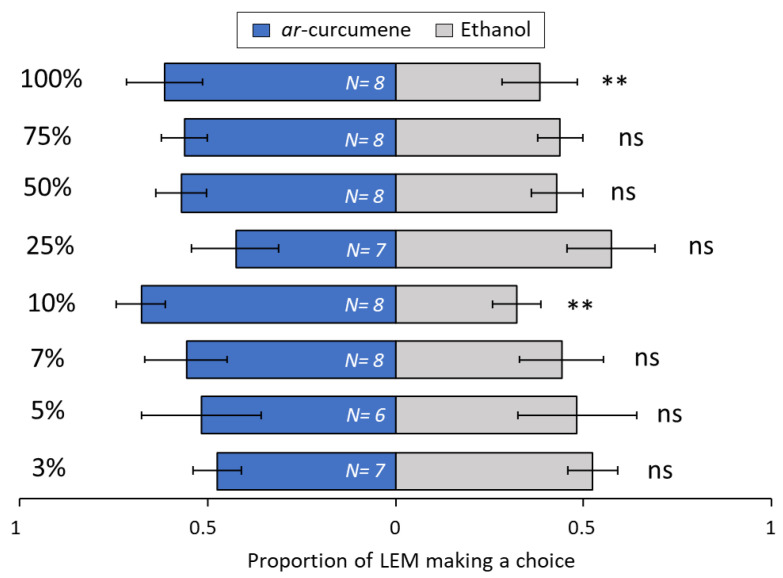
Preference of LEM to the volatile compound *ar*-curcumene and its solvent ethanol (control), in a dual-choice experimental unit. Panel shows all eight ar-curcumene concentrations tested: 3%, 5%, 7%, 10%, 25%, 50%, 75% and 100%. Numbers inside bars represent number of replicates; GLMM: ** *p* < 0.01; ns, not significant. Bars indicate proportion (±SE) of mites reaching the end section of the slide towards *ar*-curcumene and ethanol in 24 h.

**Figure 10:**
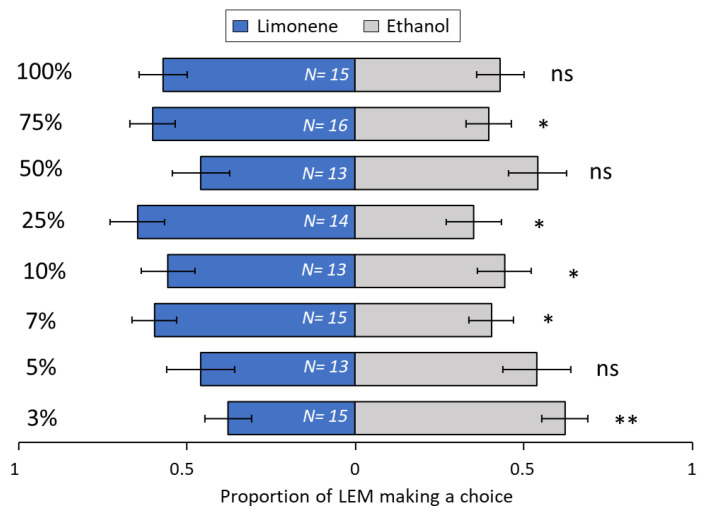
Preference of LEM to the volatile compound limonene and its solvent ethanol (control), in a dual-choice experimental unit. Panel shows all eight Limonene concentrations tested: 3%, 5%, 7%, 10%, 25%, 50%, 75% and 100%. Numbers inside bars represent number of replicates; GLMM: * *p* < 0.05; ** *p* < 0.01; ns, not significant. Bars indicate proportion (±SE) of mites reaching the end section of the slide towards limonene and ethanol in 24 h.

**Figure 11:**
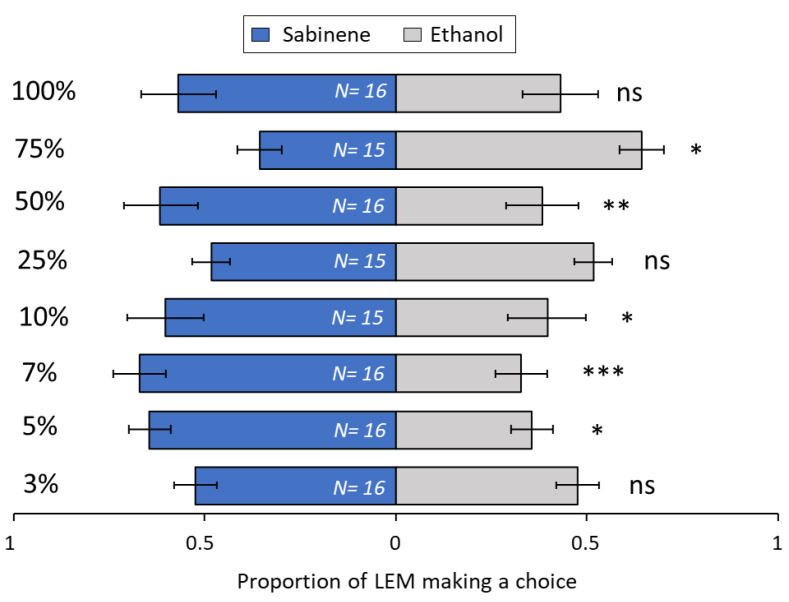
Preference of LEM to the volatile compound sabinene and its solvent ethanol (control), in a dual-choice experimental unit. Panel shows all eight Sabinene concentrations tested: 3%, 5%, 7%, 10%, 25%, 50%, 75% and 100%. Numbers inside bars represent number of replicates; GLMM: * *p* < 0.05; ** *p* < 0.01, *** *p* < 0.001; ns, not significant. Bars indicate proportion (±SE) of mites reaching the end section of the slide towards sabinene and ethanol in 24 h.

Among the terpenoids tested, the sesquiterpene β-caryophyllene (Figure 8) was very attractive to LEM in lower concentrations, at 3% (*p* = 0.008), 5% (*p* = 0.01) and 7% (*p* ≤ 0.001), but attractiveness was lost with increased concentrations, i.e., 10% (*p* = 0.19), 25% (*p* = 0.69), 50% (*p* = 0.36), 75% (*p* = 0.34) and 100% (*p* = 0.09). For the sesquiterpene *ar*-curcumene (Figure 9), only two concentrations were attractive to LEM, 10% (*p* = 0.001) and 100% (*p* = 0.002). All other concentrations were not attractive: 3% (*p* = 0.27), 5% (*p* = 0.1), 7% (*p* = 0.1), 25% (*p* = 0.27), 50% (*p* = 0.1) and 75% (*p* = 0.31). The monoterpene limonene (Figure 10) at 3% concentration (*p* = 0.008) was repellent to LEM i.e., more mites chose EtOH over the limonene. By contrast, three concentrations, 10% (*p* = 0.04), 25% (*p* = 0.05) and 75% (*p* = 0.01), were attractive to LEM, while all other tested concentrations were not attractive nor repellent: 5% (*p* = 0.10), 7% (*p* = 0.10), 50% (*p* = 0.69) and 100% (*p* = 0.14). For the monoterpene sabinene (Figure 11), the concentrations of 5% (*p* = 0.03), 7% (*p* ≤ 0.001), 10% (*p* = 0.02) and 50% (*p* = 0.003) were attractive, the concentration of 75% was repellent (*p* = 0.02), and the concentrations 3% (*p* = 0.28), 25% (*p* = 0.71) and 100% (*p* = 0.47) were not attractive nor repellent. When given a choice between same odor sources in both ends of the slide (clean vs. clean or ethanol vs. ethanol), there was no significant difference in the number of LEM that reached each source (*p* = 0.15 and *p* = 1, respectively) (Figure S3).

## 4. Discussion

Using HS-SPME collections and GC-MS analyses, 58 volatile compounds were identified from lychee, of which 44 were terpenoids (Table 1). There were large quantitative and qualitative differences in the blend of volatiles released from distinct plant tissues (B1–B4) and among plants with varying LEM infestation levels (A1–A5). Uninfested field-collected samples obtained from floral buds (B1), open flowers (B2), leaf buds (B3), and new leaf shoots (B4) had 30 unique compounds, mostly terpenoids (Table 1, Figure 2), that were not detected in LEM infested lychee plants. The PCA analysis applied based on the GC-MS data from samples B1 to B4 showed that these plant tissues can be clustered in two groups (Figure 4). The sesquiterpenes α-zingiberene, *ar*-curcumene and β-caryophyllene were the most important volatiles separating floral buds (B1), leaf buds (B3) and new leaf shoots (B4) from the open flowers (B2). Although sesquiterpene hydrocarbons were the most abundant compounds detected in these plant tissues, the monoterpene alcohol linalool and its *cis*- and *trans*-linalool oxides were only detected in open flowers (B2). The monoterpene hydrocarbons α-thujene, α-pinene, sabinene and (E)-β-ocimene were present in higher quantities in the floral buds (B1) and open flowers (B2) than in the other plant tissues. The sesquiterpene hydrocarbons β-caryophyllene and α-zingiberene were mostly detected in leaf buds (B3) and new leaf shoots (B4). 

In agreement with our findings, previous studies have also found that the most abundant compounds present in lychee samples were terpenoids. Wu et al. [44] identified 43 volatiles in lychee fruit from nine lychee cultivars and found remarkable qualitative and quantitative differences among cultivars. Nonetheless, lychee cultivars held different terpene profiles that played an important role in discriminating them. For instance, only 11 terpenoids were found in ‘Xiangli’ and ‘Jizuili’, while ‘Guangxi Huaizhi’ and ‘Guangdong Huaizhi’ had 30 and 27 terpenoids, respectively. Linalool, *cis*-rose oxide, α-terpineol, β-citronellol, geraniol, and *p*-cymene were identified in all lychee cultivars, while α-copaene and *cis*-pyran linalool oxide were found only in ‘Nuomici’ and ‘Guiwei’, respectively. Besides terpenoids, they also found ester aldehydes and alcohols in less abundance. By using a solvent-assisted flavor evaporation (SAFE) coupled with gas chromatography-olfactometry/mass spectrometry (GC-O/MS), Feng et al. [45] identified 31 volatiles in lychee fruit with methional, geraniol, furaneol, nerol and linalool pointed as the most abundant compounds. Similarly, Chyau et al. [52] reported alcohols and terpenoids as major components of lychee, and Sivakumar et al. [53] reported that nearly all the volatiles from lychee fruit cv. Mauritius and McLean’s Red were terpenoids or derivatives. Nerol, citronellol, geraniol, linalool, limonene, terpinolene, myrcene, germacrene D, β-ocimene, β-myrcene, α-muurolene and curcumene were the compounds identified as responsible for the characteristic odor of ‘Mauritius’ fruit [47,53]. By using simultaneous distillation extraction (SDE), Wang et al. [48] extracted 13 terpenoids, 3 aldehydes, 1 acid, 2 alcohols and 1 ester from young and old lychee leaves. Major terpenoids found were zingiberene, trans-caryophyllene, α-sesquiphellandrene, α-humulene, α-bergamotene and α-copaene. The terpenoids δ-elemene and L-linalool were only detected in young leaves.

In our subsequent experiment using potted plants (A1–A5), 14 unique compounds were found, mostly aldehydes and alkanes (Table 1, Figure 2). In the PCA analysis from samples A1 to A5, the abundant aldehydes nonanal and decanal were found to have a strong discrimination effect among the LEM infestation levels, separating them into two clusters (Figure 3). The first cluster included the initial infestation (A1), heavy infestation (A3) and uninfested plant (A5) treatments, and the second cluster included the intermediate infestation (A2) and overexploitation (A4) treatments. In addition, the terpenoids limonene, β-caryophyllene, *ar*-curcumene and α-zingiberene also contributed greatly to discriminate among LEM infestation levels. Hence, the terpenoids β-caryophyllene, *ar*-curcumene and α-zingiberene were found to play an important role in the discrimination among both the infested and uninfested lychee plants (A), besides their participation in the discrimination among the field-collected tissues (B). Importantly, while the levels of the most abundant aldehydes (nonanal, decanal) decreased with the progression of the LEM infestation, the most abundant terpenoids (sabinene, limonene, *ar*-curcumene and β-caryophyllene) and alkanes (tetradecane, hexadecane, heptadecane) increased in the presence of LEM, suggesting that those major compounds may play an important role in lychee plant defense and LEM attraction/repellence for these compounds might differ. 

Recently, Gunpal and Patni [54] showed that lychee leaflets infested by LEM were rich in sugars, terpenoids, linoleic acid esters, vitamins, antioxidants, glycerol, and steroids. Our results were different from those reported by Gunpal and Patni [54] and we attribute these differences to the sampling methods. They used dried, powdered leaves extracted by Soxhlet apparatus with methanol. Our analyses using the HS-SPME method possess unsurpassed sensitivity and specificity in volatile sampling. In addition, we used authentic standards to confirm the identity of the compounds, while Gunpal and Patni [54] based their identification solely on the mass spectra of peaks. Second, our study offers further knowledge, demonstrating for the first time that the volatile profile of LEM infested lychee plants changes according to LEM infestation level. It has been shown by Popitanu et al. [71] that the erineum-forming mite *Aceria erinea* induced significant emissions of terpenoids in Persian walnut leaves (*Juglans regia*) and these emissions scaled positively with the percentage of the infested leaf area. In agreement with these findings, our study showed that terpenoids correlated positively with the LEM progression of infestation. These findings raised the hypothesis that LEM responses to the main terpenoids emitted by lychee are dose dependent. Aiming to address this question, we designed a dual-choice arena (Figure 1) to investigate the LEM preferences when offering the terpenoids β-caryophyllene, *ar*-curcumene, limonene and sabinene on different concentrations. In fact, we confirmed our hypothesis by showing that key terpenoids played an important role in LEM attraction or repellence, depending on their concentration (Figure 8, Figure 9, Figure 10 and Figure 11).

Terpenoid VOCs play an important role in direct and indirect plant defenses [3]. These compounds can be toxic and repellent to the attacking insects [72] and mites [73], and can attract natural enemies of herbivores [72,74]. For example, the terpenoids α-pinene, eugenol, limonene, and terpinolene repel several mosquito species [75,76], and α-pinene repels the tick *Ixodes ricinus* [77]. Additionally, the sesquiterpene β-caryophyllene has been shown to repel mosquitoes [78] and the Asian citrus psyllid, *Diaphorina citri* Kuwayama (Hemiptera: Liviidae) [79]. Terpenoids can also act as elicitors that trigger plant defense mechanisms to target pests. For instance, tomato plants exposed to the volatiles of transgenic tobacco enriched in β-ocimene decreased aphid, *Macrosiphum euphorbiae*, development and reproduction (direct defense) and increased attraction of its natural enemy, the parasitoid *Aphidius ervi* (indirect defense) [72]. Similarly, by inhibiting the emission of terpenoid HPIVs, Mumm et al. [74] demonstrated that the inhibition of those terpenoids in mite-infested plants decreased the attractiveness of the predatory mite *Phytoseiulus persimilis*. 

Like terpenoids, aldehydes can also act as specific elicitor molecules emitted after herbivory, pathogen infection, or oxidative stresses [80,81]. For example, Yi et al. [81] showed that Lima bean plants growing adjacent to plants chemically induced with benzothiadiazole up-regulated the production of nonanal and were more resistant to infection by the pathogen *Pseudomonas syringae*. In insect-plant systems, infestation by the peach aphid (*Myzus persicae*) and the Colorado potato beetle (CPB), (*Leptinotarsa decemlineata*) drastically increased the amounts of nonanal in attacked plants [82]. The increased amounts of nonanal in potato plants infested with CPB played an important role in the attraction of the two predators, *Podisum maculiventris* and *Perillus bioculatus* [80]. However, nonanal has also been shown to increase the attraction and oviposition of *Helicoverpa assulta* (Lepidoptera: Noctuidae) moths in tobacco plants [83]. In the current study, the emissions of nonanal and decanal decreased with increasing LEM infestation (Table 1). Although the results from the behavioral assays were inconsistent with increasing doses of nonanal and decanal (Figure 6 and Figure 7), in the trials with lower concentrations of decanal (3, 5, and 7%) the treated-side was more attractive. Taken together, these data may suggest that decanal may initially elicit attraction to LEM, and as infestation progresses and decanal emissions increase, infested tissues may become less attractive to LEM. 

The attractiveness of volatile odors to most insects and mites is dose dependent [84,85,86,87]. Similarly, in the present study, we found that the compound concentration was the most important factor determining attraction or repellence of LEM. A trend towards attraction of LEM at low and intermediate concentration and repellence at high concentration was found in three out of the six volatiles tested. Decanal (Figure 7), β-caryophyllene (Figure 8) and limonene (Figure 10) were mostly attractive to LEM at low and intermediate concentrations. Sabinene (Figure 11) promoted attraction of LEM in almost all concentrations tested, and nonanal (Figure 6) promoted repellence or had no effect on LEM preferences. Importantly, since we tested individual compounds, the outcome might be different when testing them in a mixture. Further research should investigate if volatile emissions of these compounds are associated with natural enemies of LEM.

Several studies have shown that the ratio of odor blends are important in insect attraction [88,89,90]. Blends of volatiles in the proper ratios rather than single compounds can mimic natural conditions. Thus, the next step in understanding the attractive role of the lychee volatiles identified in this study to LEM is testing different odor blends at different ratios. Nevertheless, the findings that the LEM in this study significantly preferred some volatile compounds at lower concentrations is an important first step in understanding the attractive behavior of this pest. Further, to our knowledge, this is the first description of a bioassay for testing LEM odor preferences. It is important to note that because LEM hides in the erineum it was not possible to assess the number of mites released in the arena. In an erineum disc of 0.5 mm dimeter the LEM population may vary from 0 to more than 8000 mites. This information, however, can only be confirmed after the mites exit the erineum. In addition, most LEM cannot survive outside the erinea for more than 24 h [39], meaning that mites that did not make their decision within the period of observation (24 h), ended up dead in the middle of the arena. As this method allows mites to walk freely, it is uncertain how long a mite stayed on either side of the slide. Hence, we did not evaluate the activity of the mites, but the proportion of mites choosing one specific odor over another and whether the compound repelled or attracted those mites. By assessing only their final choice, this experimental set-up might have decreased the chances of having undecided mites influencing the results. One may wonder why there was a variation in LEM response when offered the choice between leaf shoot vs. leaf shoot (control group). The non-infested lychee tissue used was field-collected. Individual lychee trees may be exposed to different biotic and abiotic conditions that in turn may influence nutrition or synthesis of defensive compounds. These factors can also affect mite behavior. In our study we were not able to control for this variation.

Finally, LEM is a host specific eriophyid mite known to attack several lychee tissues [32]. Consistent with what has been observed in the field, here we showed that LEM’s most preferred tissue were the new leaf shoots (Figure 5). The attacked young tissue and the erinea progress simultaneously, meaning that the infested old plant tissue only dies months after exploitation by the mite (Ataide et al. *in preparation*). Once new growth starts to form, mite infestation in new tissue prevails and soon the whole plant is infested. This is important information for the management of LEM. It is known that lychee trees undergo two to three waves of flushing each year, usually in the summer and early in the autumn in southern China [91]. Thus, this is a critical period for prophylactic treatments with pesticides. Once the new leaf shoots are protected, LEM spread within and between plants and therefore yield losses are expected to be suppressed.

Here, we showed that there is the potential to use key terpenoids and aldehydes in the attraction or repellence of LEM to lychee plants. In the future, lychee VOCs or HIPV can be used to develop volatile-based management strategies against LEM. HIPVs, especially elicitors such as the plant hormones salicylic acid and jasmonic acid, could induce the plant defense system before insect attack [91,92,93,94,95]. In addition, a “semiochemically assisted trap cropping”, which consists of the use of trap crops supplemented with lures, such as pheromones or kairomones, can enhance the effectiveness of pest control [96]. All these strategies have contributed to the detection, monitoring, and manipulation of populations of native and invasive insect species around the world. However, these strategies have been barely investigated for mite control [97] and there are still significant knowledge gaps in the chemical communication in the acari compared to insects. As they are wingless organisms, we envision a management system for LEM focused on the use of attractant semiochemicals to lure and target them outside the erinea. Alternatively, repellents could potentially be used to protect new shoots from LEM infestation. Overall, by addressing LEM preferences to plant volatiles and specific plant tissues, we hope that new control strategies can be developed.

## Figures and Tables

**Table 1:** Percent (%) composition of volatile emissions from potted (infested) lychee plants and from field-collected (non-infested) lychee tissues (A1–A5; B1–B4).

#	* RRI_exp_	** RRI_lit_	Compounds	(Mean ± SE), *n* = 9					(Mean ± SE), *n* = 3				*** IM
				A1	A2	A3	A4	A5	B1	B2	B3	B4	
1	856	859	(*Z*)-3-hexen-1-ol	-	-	-	-	-	0.16 ± 0.06	-	-	0.25 ± 0.25	MS, RI, Std
2	927	930	α-thujene	0.27 ± 0.16	0.71 ± 0.69	-	-	-	1.71 ± 1.05	1.32 ± 1.22	-	-	MS, RI
3	933	939	α-pinene	0.70 ± 0.50	1.96 ± 1.84	0.30 ± 0.30	3.73 ± 2.40	0.09 ± 0.05	1.99 ± 1.17	1.45 ± 1.35	-	0.02 ± 0.02	MS, RI, Std
4	972	975	sabinene	5.29 ± 3.25	5.56 ± 4.54	1.92 ± 1.92	11.67 ± 4.94	4.50 ± 2.25	6.49 ± 2.97	4.23 ± 0.69	0.18 ± 0.10	0.20 ± 0.11	MS, RI, Std
5	980	979	β-pinene	-	-	-	-	-	-	0.43 ± 0.33	-	-	MS, RI, Std
6	983	985	6-methyl-5-hepten-2-one	-	-	-	-	-	0.07 ± 0.06	-	0.01 ± 0.01	0.12 ± 0.12	MS, RI, Std
7	1005	1002	(*E*)-3-hexenyl acetate	-	-	-	-	-	0.54 ± 0.27	-	-	0.03 ± 0.03	MS, RI
8	1016	1013	(*E*)-2-hexenyl acetate	-	-	-	-	-	0.05 ± 0.04	-	-	0.02 ± 0.02	MS, RI
9	1027	1029	limonene	7.11 ± 2.88	3.81 ± 1.24	7.94 ± 4.77	4.00 ± 1.28	4.56 ± 1.72	0.02 ± 0.01	0.11 ± 0.10	-	0.02 ± 0.02	MS, RI, Std
10	1046	1050	(*E*)-β-ocimene	0.68 ± 0.48	2.00 ± 1.65	0.78 ± 0.32	0.04 ± 0.04	1.54 ± 1.08	0.59 ± 0.30	5.74 ± 1.14	0.03 ± 0.02	0.40 ± 0.20	MS, RI, Std
11	1057	1059	γ-terpinene	0.85 ± 0.44	0.83 ± 0.83	0.48 ± 0.48	1.46 ± 1.46	0.27 ± 0.21	0.98 ± 0.54	0.50± 0.25	-	-	MS, RI, Std
12	1071	1072	*cis*-linalool oxide (*cis*-furanoid)	-	-	-	-	-	-	0.74 ± 0.38	-	-	MS, RI, Std
13	1086	1086	*trans*-linalool oxide (*trans*-furanoid)	-	-	-	-	-	-	3.74 ± 1.90	-	-	MS, RI, Std
14	1088	1088	terpinolene	-	0.10 ± 0.10	-	0.24 ± 0.24	-	-	-	-	-	MS, RI, Std
15	1100	1096	linalool	-	-	-	-	-	-	1.72 ± 0.31	-	-	MS, RI, Std
16	1101	1100	nonanal	43.52 ± 8.13	24.06 ± 8.19	29.31 ± 8.17	18.65 ± 6.60	51.52 ± 8.97	-	-	-	-	MS, RI, Std
17	1165	1174	*cis*-linalool oxide (*cis*-pyranoid)	-	-	-	-	-	0.01 ± 0.00	0.11 ± 0.10	-	-	MS, RI
18	1170	1176	*trans*-linalool oxide (*trans*-pyranoid)	-	-	-	-	-	0.01 ± 0.00	1.15 ± 0.58	-	-	MS, RI
19	1194	1196	estragole	0.10 ± 0.10	-	-	-	-	-	-	-	-	MS, RI, Std
20	1202	1201	decanal	6.74 ± 2.27	2.83 ± 1.40	6.11 ± 2.03	2.28 ± 1.00	7.76 ± 2.66	-	-	-	-	MS, RI, Std
21	1255	1263	(*E*)-2-decanal	1.58 ± 0.83	0.45 ± 0.30	0.27 ± 0.20	0.48 ± 0.25	-	-	-	-	-	MS, RI
22	1300	1306	undecanal	0.30 ± 0.18	0.12 ± 0.08	0.18 ± 0.10	0.12 ± 0.07	0.28 ± 0.12	-	-	-	-	MS, RI
23	1330	1338	δ-elemene	-	-	-	-	-	0.01 ± 0.00	-	-	0.03 ± 0.03	MS, RI
24	1342	1348	α-cubebene	-	-	-	-	-	0.16 ± 0.12	0.22 ± 0.21	0.42 ± 0.02	0.08 ± 0.08	MS, RI
25	1353	1360	(*E*)-2-undecanal	0.12 ± 0.09	0.09 ± 0.09	-	0.06 ± 0.04	-	-	-	-	-	MS, RI
26	1359	1371	cyclosativene	-	-	-	-	-	1.15 ± 0.28	0.63 ± 0.33	1.47 ± 0.09	1.00 ± 0.02	MS, RI, Std
27	1369	1375	α-ylangene	-	-	-	-	-	0.11 ± 0.07	0.18 ± 0.17	0.18 ± 0.09	0.10 ± 0.03	MS, RI
28	1367	1376	α-copaene	1.08 ± 1.05	1.73 ± 0.51	2.82 ± 0.85	1.08 ± 0.26	2.23 ± 1.50	2.39 ± 0.36	2.04 ± 0.26	2.86 ± 0.16	2.44 ± 0.28	MS, RI, Std
29	1382	1391	7-*epi*-sesquithujene	0.94 ± 0.62	3.69 ± 0.79	1.44 ± 0.59	2.82 ± 0.52	1.90 ± 0.38	6.30 ± 0.91	6.02 ± 1.06	6.45 ± 0.07	7.32 ± 1.54	MS, RI
30	1389	1400	sibirene	-	-	-	-	-	0.07 ± 0.03	0.30 ± 0.29	-	0.02 ± 0.02	MS, RI
31	1390	1400	tetradecane	3.30 ± 1.35	2.16 ± 0.68	1.04 ± 0.38	1.37 ± 0.67	1.85 ± 1.08	-	-	-	-	MS, RI, Std
32	1392	1403	methyl eugenol	1.41 ± 1.16	-	-	-	-	-	-	-	-	MS, RI, Std
33	1394	1405	sesquithujene	-	-	-	-	-	0.03 ± 0.02	0.20 ± 0.10	0.05 ± 0.05	0.03 ± 0.03	MS, RI
34	1396	1408	dodecanal	0.20 ± 0.11	0.10 ± 0.05	0.16 ± 0.09	0.04 ± 0.04	0.02 ± 0.02	-	-	-	-	MS, RI
35	1410	1419	β-caryophyllene	5.69 ± 3.08	8.10 ± 1.83	10.34 ± 2.89	6.23 ± 1.72	5.21 ± 2.12	12.60 ± 3.69	10.57 ± 0.81	13.98 ± 0.61	13.32 ± 1.53	MS, RI, Std
36	1411	1420	β-cedrene	-	-	-	-	-	1.04 ± 0.29	0.52 ± 0.42	1.25 ± 0.06	0.55 ± 0.32	MS, RI
37	1423	1432	*trans*-α-bergamotene	0.60 ± 0.26	1.45 ± 0.39	1.13 ± 0.58	1.16 ± 0.28	0.56 ± 0.25	1.75 ± 0.15	1.15 ± 0.19	2.15 ± 0.06	1.58 ± 0.16	MS, RI
38	1428	1441	aromadendrene	-	-	-	-	-	0.13 ± 0.12	0.09 ± 0.08	0.32 ± 0.11	0.27 ± 0.14	MS, RI, Std
39	1431	1444	6,9-guaiadiene	0.02 ± 0.02	0.04 ± 0.04	0.02 ± 0.02	0.01 ± 0.01	-	-	-	-	-	MS, RI
40	1435	1450	*cis*-muurola 3,5-diene	-	-	-	-	-	0.11 ± 0.10	0.30 ± 0.29	0.21 ± 0.06	0.20 ± 0.11	MS, RI
41	1439	1454	α-humulene	-	-	-	-	-	1.66 ± 0.82	0.64 ± 0.54	2.19 ± 0.19	1.45 ± 0.24	MS, RI, Std
42	1442	1456	(*E*)-β-farnesene	-	-	-	-	-	0.50 ± 0.23	0.71 ± 0.33	1.43 ± 0.08	0.54 ± 0.27	MS, RI
43	1448	1463	*cis*-cadina-1(6),4-diene	-	-	-	-	-	0.07 ± 0.06	0	0.05 ± 0.04	0.07 ± 0.04	MS, RI
44	1453	1466	α-acoradiene	-	-	-	-	-	0.05 ± 0.02	0.35 ± 0.34	0.03 ± 0.02	0.06 ± 0.06	MS, RI
45	1462	1476	trans-cadina-1(6),4-diene	-	-	-	-	-	0.64 ± 0.29	0.56 ± 0.35	1.15 ± 0.06	0.55 ± 0.28	MS, RI
46	1466	1479	γ-muurolene	-	-	-	-	-	7.36 ± 4.41	4.85 ± 1.73	4.26 ± 0.68	7.70 ± 4.98	MS, RI
47	1468	1480	*ar*-curcumene	10.25 ± 3.18	32.51 ± 6.4	30.33 ± 6.38	38.54 ± 5.22	13.27 ± 3.97	16.78 ± 8.30	26.50 ± 4.07	11.64 ± 3.39	21.64 ± 5.33	MS, RI, Std
48	1476	1485	germacrene D	-	-	-	-	-	0.05 ± 0.04	-	-	-	MS, RI
49	1483	1493	α-zingiberene	0.24 ± 0.12	2.03 ± 0.77	1.50 ± 0.79	1.72 ± 0.49	1.34 ± 0.40	27.65 ± 4.89	16.50 ± 0.74	39.62 ± 1.43	33.73 ± 7.23	MS, RI
50	1486	1500	pentadecane	0.82 ± 0.45	1.03 ± 0.48	0.21 ± 0.12	0.37 ± 0.19	0.48 ± 0.32	-	-	-	-	MS, RI, Std
51	1489	1500	α-muurolene	-	-	-	-	-	0.86 ± 0.27	0.66 ± 0.36	1.01 ± 0.14	1.24 ± 0.06	MS, RI
52	1493	1505	(*E*,*E*)-α-farnesene	-	-	-	-	-	0.56 ± 0.10	0.99 ± 0.43	0.84 ± 0.02	0.60 ± 0.09	MS, RI, Std
53	1500	1515	β-curcumene	-	-	-	-	-	0.78 ± 0.31	0.37 ± 0.27	0.92 ± 0.10	0.21 ± 0.03	MS, RI
54	1507	1522	β-sesquiphellandrene	-	-	-	-	-	3.46 ± 0.97	2.02 ± 0.15	5.94 ± 0.04	3.13 ± 0.56	MS, RI
55	1509	1523	δ-cadinene	0.11 ± 0.07	1.12 ± 0.39	0.63 ± 0.35	2.03 ± 0.45	0.17 ± 0.10	0.10 ± 0.00	0.10 ± 0.00	0.71 ± 0.05	0.03 ± 0.01	MS, RI
56	1547	1561	germacrene B	-	-	-	-	-	0.07 ± 0.06	0.34 ± 0.24	0.12 ± 0.07	0.13 ± 0.07	MS, RI
57	1586	1600	hexadecane	2.21 ± 1.06	1.48 ± 0.71	0.88 ± 0.36	0.99 ± 0.52	1.29 ± 0.86	-	-	-	-	MS, RI, Std
58	1699	1700	heptadecane	5.38 ± 4.08	1.22 ± 0.81	0.85 ± 0.55	0.64 ± 0.37	0.89 ± 0.58	-	-	-	-	MS, RI, Std
			Total %	99.51 ± 0.18	99.18± 0.32	98.64 ± 0.64	99.73 ± 0.11	99.73 ± 0.14	99.06 ± 0.91	98.05 ± 0.52	99.47 ± 0.23	99.08 ± 0.43	

* RI_exp_: Retention indices (RI) calculated from the current study. ** RR_lit_: Retention indices (RI) obtained from literature source [Adams Library, 2007]. *** IM: Identification method based on mass spectra (MS); Retention indices (RI) and authentic compounds (Std) on the DB-5 column. -: Not detected. Initial infestation (A1), intermediate infestation (A2), heavy infestation (A3), overexploitation (A4), and healthy, uninfested plant (A5). Floral buds (B1), open flowers (B2), leaf buds (B3), and new leaf shoots (B4).

**Table 2:** Eigenvalues for principal components F1 to F4 (A1 to A5).

Principal Components	Eigenvalue	Proportion of Total Variance (%)	Cumulative Proportion of Total Variance (%)
F1	9.575	45.597	45.597
F2	5.852	27.868	73.465
F3	3.424	16.307	89.772
F4	2.148	10.228	100.00

**Table 3:** Relative contributions and squared cosines of each principal component to samples A1 to A5.

Variables	Contribution of the Variables (%)				Squared Cosines of the Variables *			
	F1	F2	F3	F4	F1	F2	F3	F4
α-thujene	0.059	3.461	20.992	3.395	0.006	0.203	0.719	0.073
α-pinene	5.847	6.635	1.363	0.241	0.560	0.388	0.047	0.005
sabinene	2.758	7.930	3.957	6.345	0.264	0.464	0.136	0.136
limonene	3.377	1.984	3.633	20.304	0.323	0.116	0.124	0.436
(*E*)-β-ocimene	0.075	1.052	26.445	1.193	0.007	0.062	0.906	0.026
γ-terpinene	2.717	10.184	3.936	0.424	0.260	0.596	0.135	0.009
nonanal	7.633	1.236	0.217	8.815	0.731	0.072	0.007	0.189
decanal	7.440	4.369	0.172	1.209	0.712	0.256	0.006	0.026
(*E*)-2-decanal	3.320	8.718	0.547	7.133	0.318	0.510	0.019	0.153
α-copaene	0.005	16.126	0.816	1.298	0.000	0.944	0.028	0.028
7-*epi*-sesquiterpene	6.753	1.033	7.608	1.507	0.647	0.060	0.261	0.032
tetradecane	5.307	6.955	2.478	0.000	0.508	0.407	0.085	0.000
methyl eugenol	6.544	5.017	0.560	2.819	0.627	0.294	0.019	0.061
β-caryophyllene	1.347	4.182	0.449	28.441	0.129	0.245	0.015	0.611
*trans*-aα-bergamotene	7.245	0.099	2.819	9.497	0.694	0.006	0.097	0.204
*ar*-curcumene	9.565	0.020	0.217	3.519	0.916	0.001	0.007	0.076
α-zingiberene	8.588	1.147	2.987	0.385	0.822	0.067	0.102	0.008
pentadecane	0.422	6.462	16.937	0.067	0.040	0.378	0.580	0.001
δ-cadinene	8.596	2.092	1.590	0.002	0.823	0.122	0.054	0.000
hexadecane	5.762	6.300	2.239	0.136	0.552	0.369	0.077	0.003
heptadecane	6.640	5.000	0.040	3.269	0.636	0.293	0.001	0.070
Total	100.000	100.000	100.000	100.000	9.575	5.852	3.424	2.148

* Values in bold correspond for each variable to the factor for which the squared cosine is the largest.

**Table 4:** Results of cluster analysis for samples A1 to A5.

Results by Cluster	Cluster 1	Cluster 2
Objects	A1, A3, A5	A2, A4
Sum of weights	3	2
Within-class variance	270.81	59.25
Maximum distance to centroid	17.97	5.44
Cluster centroids > 1.0	sabinene (3.90), limonene (6.54), (*E*)-β-ocimene (1.00), α-copaene (2.04), nonanal (41.45), decanal (6.87), 7-*epi*-sesquithujene (1.43), tetradecane (2.06), β-caryophyllene (7.08), *trans*-α-bergamotene (0.76), *ar*-curcumene (17.95), α-zingiberene (1.03), hexadecane (1.46), heptadecane (2.37)	α-pinene (2.84), sabinene (8.62), limonene (3.91) (*E*)-β-ocimene (1.02), γ-terpinene (1.15), nonanal (21.36), decanal (2.56), α-copaene (1.41), 7-*epi*-sesquithujene (3.26), tetradecane (1.77), β-caryophyllene (7.12), *trans*-α-bergamotene (1.31), *ar*-curcumene (35.53), α-zingiberene (1.88), δ-cadinene (1.58), hexadecane (1.24)

**Table 5:** Eigenvalues for principal components F1 to F3 (B1 to B4).

Principal Components	Eigenvalue	Proportion of Total Variance (%)	Cumulative Proportion of Total Variance (%)
F1	15.743	56.227	56.227
F2	7.018	25.065	81.292
F3	5.238	18.708	100.000

**Table 6:** Relative contributions and squared cosines of each principal component to samples B1 to B4.

Variables	Contribution of the Variables (%)			Squared Cosines of the Variables *		
	F1	F2	F3	F1	F2	F3
α-thujene	2.737	0.580	10.088	0.431	0.041	0.528
α-pinene	2.540	0.766	10.428	0.400	0.054	0.546
sabinene	2.087	1.079	11.372	0.329	0.076	0.596
(*E*)-3-hexenyl acetate	0.000	5.361	11.907	0.000	0.376	0.624
(*E*)-β-ocimene	5.340	2.221	0.066	0.841	0.156	0.003
γ-terpinene	1.412	1.709	12.555	0.222	0.120	0.658
*cis*-linalool oxide	4.916	3.121	0.136	0.774	0.219	0.007
*trans*-linalool oxide	4.916	3.121	0.136	0.774	0.219	0.007
linalool	4.916	3.121	0.136	0.774	0.219	0.007
*trans*-linalool oxide	4.944	3.072	0.114	0.778	0.216	0.006
cyclosativene	5.852	0.104	1.363	0.921	0.007	0.071
α-ylangene	0.117	13.896	0.121	0.018	0.975	0.006
α-copaene	6.123	0.499	0.019	0.964	0.035	0.001
7-*epi*-sesquithujene	1.313	4.287	9.402	0.207	0.301	0.493
β-caryophyllene	6.146	0.300	0.216	0.968	0.021	0.011
β-cedrene	3.488	0.880	7.428	0.549	0.062	0.389
*trans*-α-bergamotene	5.854	0.160	1.281	0.922	0.011	0.067
α-humulene	6.027	0.000	0.976	0.949	0.000	0.051
(*E*)-β-farnesene	2.140	9.446	0.005	0.337	0.663	0.000
*trans*-cadina-1(6),4-diene	3.593	5.625	0.756	0.566	0.395	0.040
γ-muurolene	0.000	14.161	0.118	0.000	0.994	0.006
*ar*-curcumene	5.230	0.259	3.023	0.823	0.018	0.158
α-zingiberene	6.186	0.013	0.482	0.974	0.001	0.025
α-muurolene	3.120	2.628	6.191	0.491	0.184	0.324
(*E*,*E*)-α-farnesene	1.071	11.638	0.281	0.169	0.817	0.015
β-curcumene	2.018	1.685	10.766	0.318	0.118	0.564
β-sesquiphellandrene	5.343	1.860	0.539	0.841	0.131	0.028
δ-cadinene	2.571	8.410	0.096	0.405	0.590	0.005
Total	100.000	100.000	100.000	15.743	7.018	5.238

* Values in bold correspond for each variable to the factor for which the squared cosine is the largest.

**Table 7:** Results of cluster analysis for samples B1 to B4.

Results by Cluster	Cluster 1	Cluster 2
Objects	B1, B3, B4	B2
Sum of weights	3	1
Within-class variance	84.80	0.00
Maximum distance to centroid	8.73	0.00
Cluster centroids > 1.0	sabinene (2.29), cyclosativene (1.21), α-copaene (2.56), 7-*epi*-sesquithujene (6.69), β-caryophyllene (13.30), α-humulene (1.77), γ-muurolene (6.44), *ar*-curcumene (16.69), α-zingiberene (33.67), α-muurolene (1.04),β-sesquiphellandrene (4.18)	α-thujene (1.32), α-pinene (1.45), sabinene (4.23), (*E*)-β-ocimene (5.74), *trans*-linalool oxide (3.74), linalool (1.72), *trans*-linalool oxide (1.15), α-copaene (2.04), 7-*epi*-sesquithujene (6.02), β-caryophyllene (10.57), *trans*-α-bergamotene (1.15), γ-muurolene (4.85), *ar*-curcumene (26.50), α-zingiberene (16.50), β-sesquiphellandrene (2.02)

## Data Availability

The datasets generated and analyzed during the current study are available in the figshare repository under the DOI 10.6084/m9.figshare.23269553.

## Supplementary Materials

The following supporting information can be downloaded at: https://www.mdpi.com/article/10.3390/biom13060933/s1, Figure S1: Images of the four erineum stages associated with different levels of LEM infestation. Photos of the leaflets showing the erinea were taken using an Apple iPhone 11 (f/1.8; 1/120 s; ISO-125). Photo background has been removed using Adobe^®^ Photoshop CS 5.0. Figure S2: Preference of LEM to same lychee plant tissues. Panel shows LEM choice when offered similar plant tissues in both ends of the experimental arena (leaf vs. leaf, fruit vs. fruit, flower bud vs. flower bud and leaf shoot vs. leaf shoot, *N* = 8). GLMM: * *p* < 0.05; ns, not significant. Bars indicate proportion (±SE) of mites reaching the end section of the slide (Sections 3 and 4 and TAPE) towards each plant tissue in 24 h. Figure S3: Preference of LEM to same odor sources in a dual-choice experimental unit. Panel shows LEM choice when offered same odor sources in both ends of the experimental arena (clean filter paper vs. clean filter paper, *N* = 16; ethanol vs. ethanol, *N* = 15). GLMM: ns, not significant. Bars indicate proportion (±SE) of mites reaching the end section of the slide (Sections 3 and 4 and TAPE) towards each source in 24 h.
