# Supplementary material for: Volatile Characterization of Lychee Plant Tissues (Litchi chinensis) and the Effect of Key Compounds on the Behavior of the Lychee Erinose Mite (Aceria litchii)
Source: Biomolecules. 2023 Jun 2;13(6):933. doi: 10.3390/biom13060933 (PMC10296336; doi:10.3390/biom13060933)
Supplement: Supplementary file 1 [file biomolecules-13-00933-s001.zip › biomolecules-2349190-supplementary.pdf]

Supplementary Materials

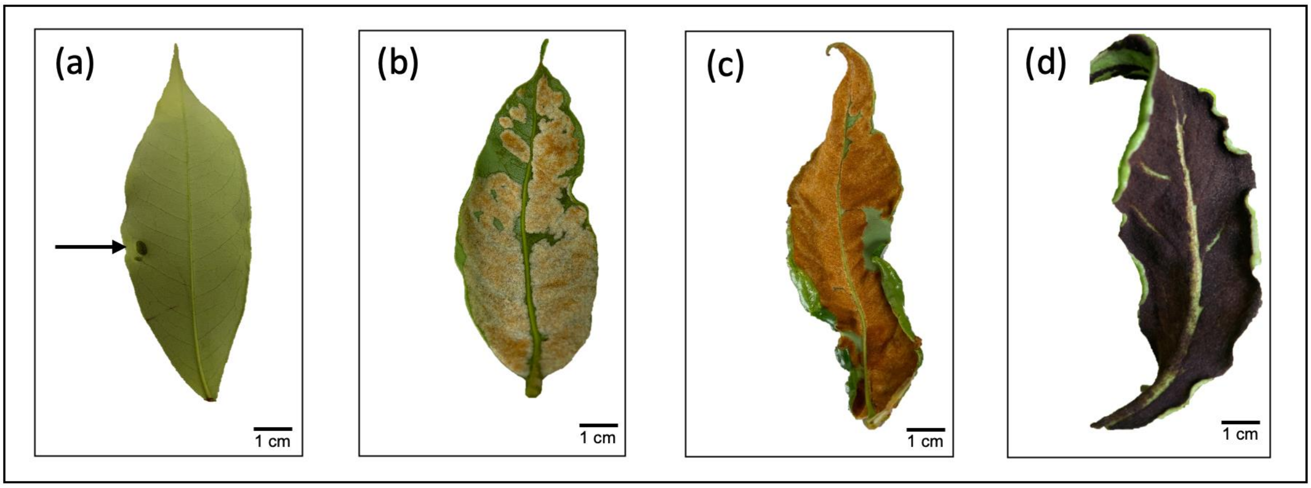

Figure S1. Images of the four erineum stages associated with different levels of LEM infestation. Photos of the leaflets showing the erinea were taken using an Apple iPhone 11 (f/1.8; 1/120 sec.; ISO-125). Photo background has been removed using Adobe® Photoshop CS 5.0.

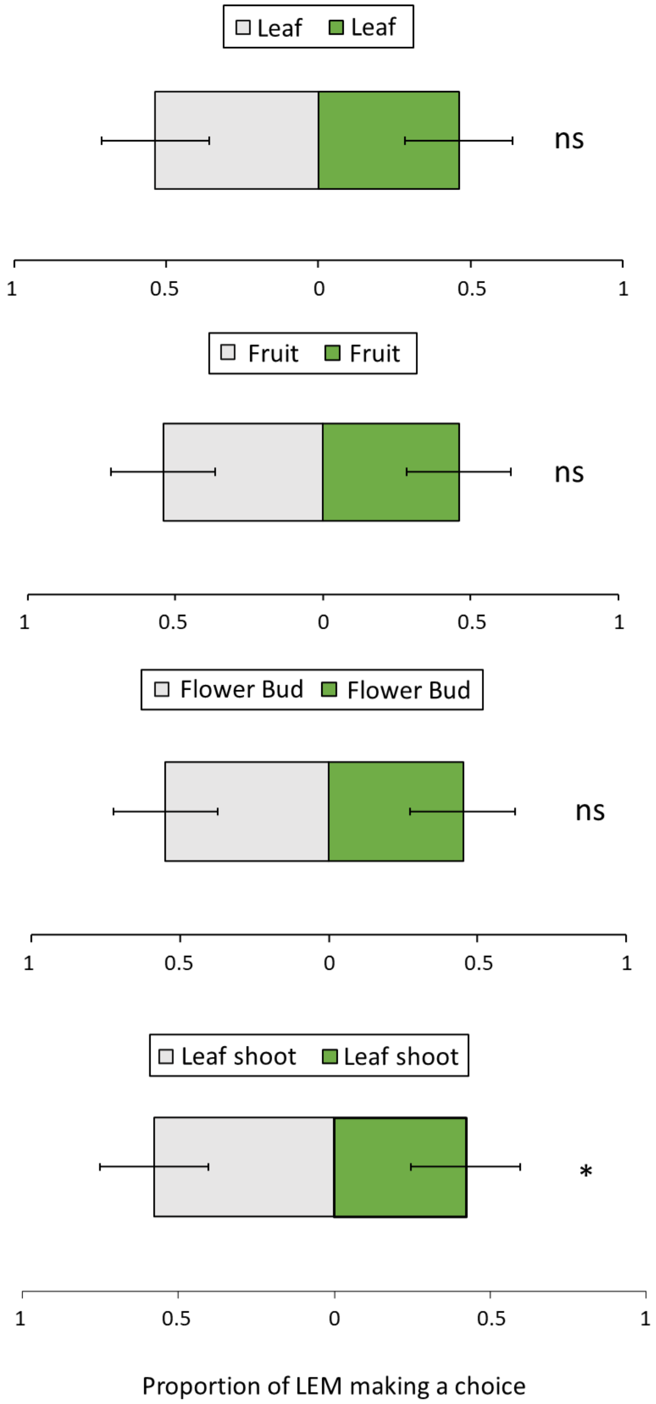

Figure S2. Preference of LEM to same lychee plant tissues. Panel shows LEM choice when offered similar plant tissues in both ends of the experimental arena (leaf vs. leaf, fruit vs. fruit, flower bud vs. flower bud and leaf shoot vs. leaf shoot, *N*= 8). GLMM: *P< 0.05; ns, not significant. Bars indicate proportion (±SE) of mites reaching the end section of the slide (sections 3, 4 and TAPE) towards each plant tissue in 24h.

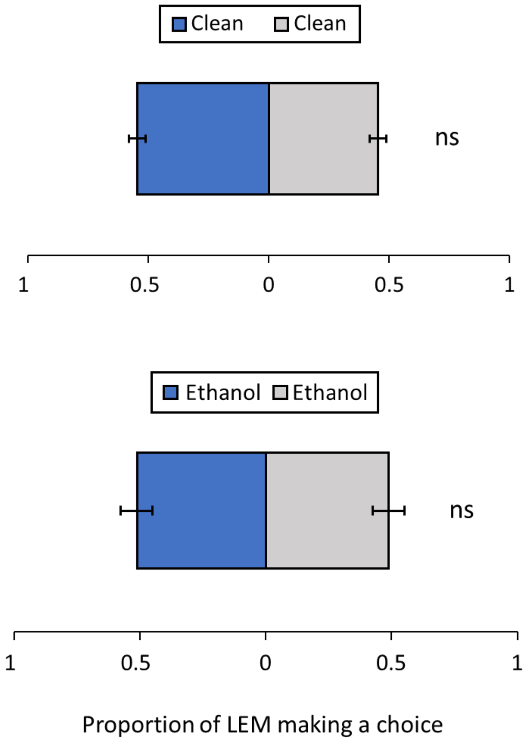

Figure S3. Preference of LEM to same odor sources in a dual-choice experimental unit. Panel shows LEM choice when offered same odor sources in both ends of the experimental arena (clean filter paper vs. clean filter paper, *N*= 16; ethanol vs. ethanol, *N*= 15). GLMM: ns, not significant. Bars indicate proportion (±SE) of mites reaching the end section of the slide (sections 3, 4 and TAPE) towards each source in 24h.
